# Virus-Like-Vaccines against HIV

**DOI:** 10.3390/vaccines6010010

**Published:** 2018-02-11

**Authors:** Anne-Marie C. Andersson, Melanie Schwerdtfeger, Peter J. Holst

**Affiliations:** Department of Immunology and Microbiology (ISIM), Faculty of Health Sciences, University of Copenhagen, The Panum Institute Building 7-13-16, Blegdamsvej 3B, DK2200 Copenhagen, Denmark; anmaca.andersson@gmail.com (A.-M.C.A.); melanies@sund.ku.dk (M.S.)

**Keywords:** virus vectors, virus-like-particles, T cells, antibodies, HIV, virus-like-vaccines

## Abstract

Protection against chronic infections has necessitated the development of ever-more potent vaccination tools. HIV seems to be the most challenging foe, with a remarkable, poorly immunogenic and fragile surface glycoprotein and the ability to overpower the cell immune system. Virus-like-particle (VLP) vaccines have emerged as potent inducers of antibody and helper T cell responses, while replication-deficient viral vectors have yielded potent cytotoxic T cell responses. Here, we review the emerging concept of merging these two technologies into virus-like-vaccines (VLVs) for the targeting of HIV. Such vaccines are immunologically perceived as viruses, as they infect cells and produce VLPs in situ, but they only resemble viruses, as the replication defective vectors and VLPs cannot propagate an infection. The inherent safety of such a platform, despite robust particle production, is a distinct advantage over live-attenuated vaccines that must balance safety and immunogenicity. Previous studies have delivered VLVs encoded in modified Vaccinia Ankara vectors and we have developed the concept into a single-reading adenovirus-based technology capable of eliciting robust CD8^+^ and CD4^+^ T cells responses and trimer binding antibody responses. Such vaccines offer the potential to display the naturally produced immunogen directly and induce an integrated humoral and cellular immune response.

## 1. Introduction

HIV and, in particular, HIV-1, is one of the most important challenges to vaccine research as it evades both antibody and T cell responses and is a major global health problem, with close to 2 million annual fatalities [1]. Despite the continued absence of a marketed vaccine against either HIV-1 or HIV-2, clinical trials and non-human primate studies have suggested that meaningful levels of protection may be achievable [2,3]. With success apparently just out of reach, the development of a vaccine against HIV-1—which is the most studied of the two HIVs (94,000 vs. 5900 pubmed hits for HIV-2) and the only one discussed in this review—has been the inspiration for much research in vaccine technology, resulting in improved tools for induction of both T cell and antibody responses. Despite these important advances, live-attenuated vaccines developed more than two decades ago [4] and attenuated natural infection [5] remain the only successful approaches against virulent simian HIV homologues.

There are many reasons for the failure to develop a viable HIV vaccine, but the diversity of the circulating HIV-1 strains, high mutation rate, and rapid seeding of intracellular DNA reservoirs, which can be invisible to the immune system, are key challenges [6]. 

**Figure 1:**
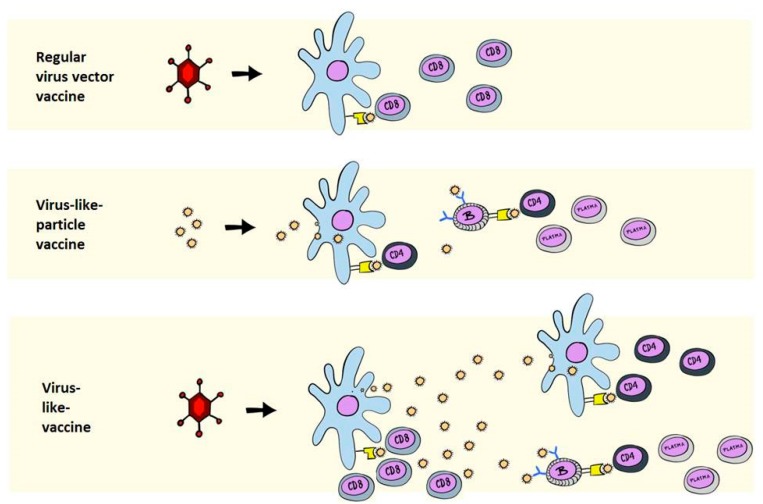
Primary immunological mechanisms of virus vectored-, VLP-, and VLV-induced antigen presentation. The top panel depicts a virus vectored vaccine that encodes an intracellular or non-secreted antigen. The principle manner of antigen presentation is via MHC class I presentation to CD8^+^ T cells through the direct presentation pathway. The middle panel depicts a classical ex vivo purified and injected VLP vaccine, which can be taken up by antigen-presenting cells to be presented on MHC class II and stimulate CD4^+^ T cells, and directly stimulates B cells with antigen in the form of VLPs. The bottom panel depicts a VLV, where the antigen is initially synthesized intracellularly for MHC class I presentation, followed by the release of VLPs, which stimulate CD4^+^ T cells and B cells, as in the middle panel.

Live-attenuated vaccines are successful, at least in part, because they maintain an activated cellular response at mucosal surfaces and mucosa draining lymph nodes, which can prevent seeding of a latent reservoir [7,8]. By being rapidly effective, they also prevent the generation of viral diversity, which would fuel subsequent immune escape. Antibody-inducing vaccines could do the same if they were effective during, or immediately following, infection and effector T cells can, as demonstrated, prevent virus spread and contain infection. However, T cells may be difficult to maintain in sufficient numbers and activation state, which points to a combined antibody- and T cell-inducing vaccine as a more realistic option [9]. But how can such an immune response be obtained? During natural acute infections with most agents (e.g., Measles), antiviral defense triggers effector mechanisms and induces life-long protective immunity following the elimination of the infection [10]. Prominent immune-mediated effector mechanisms following such an outcome are cytotoxic T cells and neutralizing antibodies, the latter being the most desired correlate of protective immunity. In HIV, the T cell response and the neutralizing antibodies arrive too late, but the demonstration of experimental efficacy by live-attenuated vaccines remains a beacon of hope in HIV vaccine research [4]. As explained, live-attenuated viral vaccines that mimic many aspects of resolved acute infection are among the most effective tools available today. However, against HIV they are considered too dangerous, and further decreasing virulence may not be possible as this would negatively affect vaccine immunogenicity and the duration of an effective memory response [11]. On the other hand, virus-like-particle (VLP) vaccines, appear to offer many of the same desirable properties as live-attenuated vaccines [12]. VLPs allow the exhibition of viral antigens on a viral surface, similar to live-attenuated vaccines, with one example being the Human Papilloma Virus (HPV) vaccines based on self-assembling papillomavirus L1 capsid proteins [13]. Whereas the HPV vaccines induce protective neutralizing antibody responses and have been translated into commercial and medical success stories, VLP-based vaccines have failed to induce protection against other diseases [14]. A notable difference between such ex vivo VLPs, natural infection, and live-attenuated vaccines, is the absence of de novo synthesis of viral antigens in vivo and, thus, only minor cytotoxic T cell responses. This is important to note as the major remaining challenges for antiviral vaccine research can all be defined as those where a relevant high-titered neutralizing antibody response cannot easily be induced. In contrast, natural immunity to infection seems to be related to the induction of protective T cell responses [15]. Therefore, a vaccine that could elicit antibodies as efficiently as VLP vaccines and simultaneously induce cytotoxic T cells could bridge natural and artificial immunity. Such vaccines can be generated by simply moving VLP production from the factory and into the vaccinee using vectored or virus-encoded VLP-based vaccines [16]. Replication-defective viral vectors encoding the machinery for producing virus particles allow such experimental vaccines to capture the essentials of a viral infection and subsequent release of VLPs. This review is dedicated to explaining how in vivo synthesized VLPs, here defined as virus-like-vaccines (VLVs) can be designed, how they compare to their parent designs VLPs and virus vectored vaccines (Figure 1, VLV principle), and, in particular, their potential in targeting HIV.

## 2. Immunological Differences between VLPs and VLVs

VLPs are highly attractive vaccine vehicles, as evidenced by their success in targeting papillomaviruses and Hepatitis B virus [12]. Initially used for homologous disease targeting, the design of chimeric VLPs has enabled the combination of non-naturally associated VLP antigens and heterologous VLP antigens to be genetically fused, “glued”, or chemically coupled to VLP vehicles [17]. The superiority of VLP-exhibited antigens, compared to antigens alone, has been shown in numerous studies [18,19,20]. For stable and simple antigens VLP antigen delivery is straightforward, with a direct translation into effective triggering of B and CD4^+^ T cell responses. Claims that VLPs can trigger CD8^+^ T cell responses are abundant in the scientific literature [21], but direct comparisons with other methods that excel in CD8^+^ T cell induction are scarce. Indeed, while VLPs improve CD4^+^ T cell and antibody responses, VLP incorporation is inferior to, and has no added effect on top of viral vector-encoded antigens [22]. This is not meant to claim that VLPs do not induce CD8^+^ T cells at all, as such responses are frequently detectable, but that there are much better ways to induce cytotoxic T cells by vaccination. 

VLVs are simply VLPs encoded within a replication deficient viral vector. Upon vaccination, the viral vector infects antigen presenting cells (APCs) to induce direct antigen presentation of the encoded antigen on MHC class I, leading to the triggering of CD8^+^ T cells [23]. Next, the infected APCs produce VLPs, which are secreted and can be presented to dendritic cells and B cells for further triggering of CD4^+^ T cells, CD8^+^ T cells, and antibody responses [16,23]. Compared to VLVs, VLPs generally contain scant genetic material and only induce limited innate immune activation, but can be engineered to increase such activation by including TLR ligands or other adjuvants [24]. Compared to DNA-encoded VLPs, viral delivery differs in the induction of more innate inflammation, the efficient direct targeting of dendritic cells, and the delivery of excess vector-derived antigen, which may contain helper T cell epitopes. The latter, admittedly, comes with a *caveat*, as these vector epitopes are also generally off-target with regard to the desired vaccine-induced protection (discussed in detail in Fougeroux et al. [25]). Overall, several key properties of individual types of VLVs, including strengths and limitations, follow the vector type used to encode VLPs, but there are differences during repeated administration that will be discussed in the following sections [26,27]. 

Another important difference between VLVs and VLPs is that VLPs require stability of both particles and surface antigens, both with an acceptable shelf-life. VLVs only require that the delivery vehicle is producible and stable, that the actual appropriately folded immunogenic antigens are produced in situ, and that the recombinant antigen does not need much stability, as it does not require any kind of storage. 

## 3. Poxviral Vectors Encoding HIV and SIV VLPs

Natural immunity and vaccine immune correlates of protection against HIV are not well defined and will be discussed in more detail in separate sections below. However, in very broad terms, control of natural HIV infection is clearly T cell-mediated [15] and artificial immunity can clearly be provided by antibodies [28]. For these reasons, it has been natural for us to develop vaccines against HIV with encoded delivery platforms capable of mobilizing both arms of the immune system. The first VLV designed to target HIV encoded inactivated HIV or SIV genomes in vaccinia or modified vaccinia Ankara vectors (MVA) [29]. This was possible because poxviral vectors are intrinsically capable of delivering large transgenic sequences. By deleting parts of the genes, viruses were made which encoded the majority of HIV and were, indeed, capable of secreting VLPs [30]. These vaccines are among the most successful pre-clinical vaccine candidates targeting HIV. When tested in human trials they induced non-neutralizing antibody responses not unlike those that elicit partial protection in non-human primate models of HIV infection [30]. Notably, a later designed recombinant canarypox vector expressing HIV Gag and protease from clade B, and gp120 of clade E fused to the transmembrane portion of HIV Env from clade B, in effect dangling gp120 monomers secreted on Gag-based VLPs, was the priming vaccine in the now famous RV144 trial in Thailand, where partial protection was induced against HIV [2].

Unfortunately, while MVA vaccines, even without a DNA prime, are excellent antibody and CD4^+^ T cell response inducers, they are not the principal inducers of CD8^+^ T cells [31]. In contrast, adenovirus vaccines are excellent inducers of CD8^+^ T cells and antibodies. Hence, it is not surprising that the combination of adenovirus and MVA in prime-boost regimens is among the most potent immunization regimens tried in humans or non-human primate (NHP) models [3,32,33]. This realization provided our rationale for designing a VLV based on adenovirus vectors, with the initial aim to combine such adenoviral vaccines with already available vaccines based on MVA. 

## 4. A Simplified Approach to the Generation of Adenovirus-based VLVs

**Figure 2:**
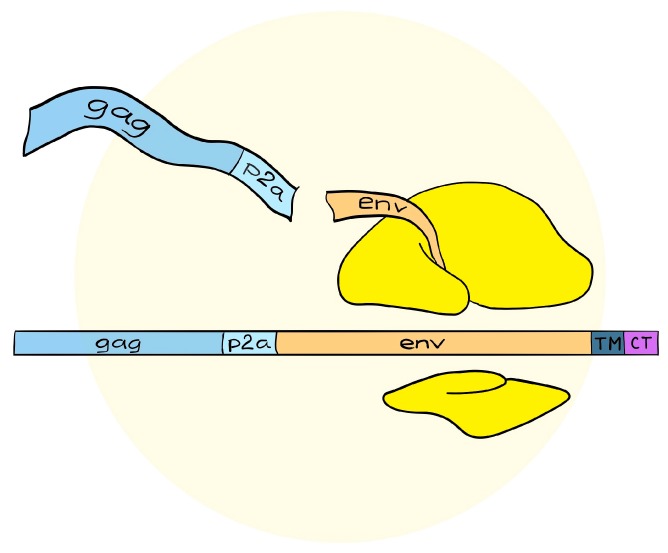
Design of the single frame transgenic cassette in adenoviral VLVs. The viral VLP-forming Gag protein is synthesized in-frame with the Env antigen, but separated with a self-cleavable peptide. This produces Gag and Env or any other downstream antigen in a 1:1 ratio.

**Figure 3:**
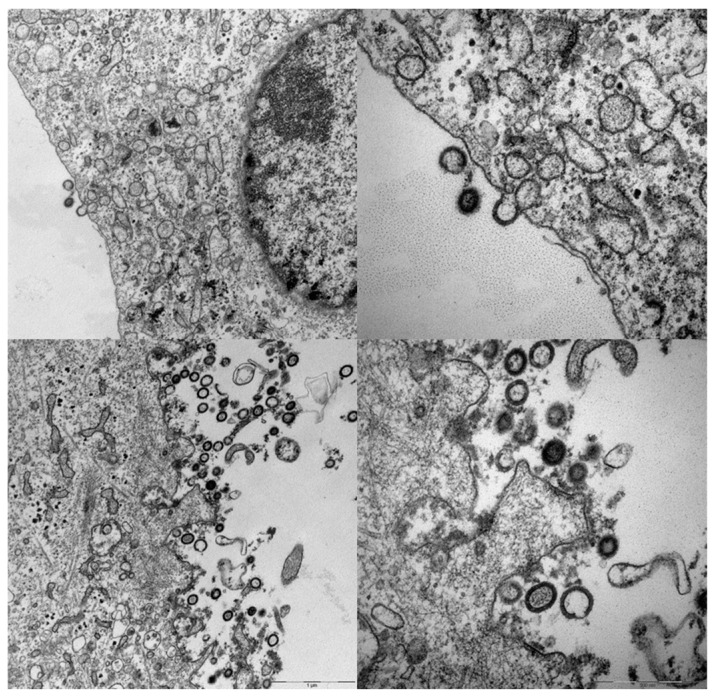
Secretion of VLPs from vector transduced cells. Vero cells infected with VLVs, then fixed, embedded, sectioned, and stained for transmission electron microscopy. The right-side micrographs depict higher magnification of the overview images to the left. The scale bar is visible at the lower right of the bottom micrographs. Specific experimental conditions were as described in Andersson et al. 2016 [7].

Compared to poxviruses, first-generation adenoviruses have a more limited cloning capacity of 4–8 kilobases, which necessitates a simplified expression cassette. In order to encode the necessary lentiviral elements, we used the self-cleavable P2A sequence, derived from Porcine Teschovirus, and encoded *gag*, followed by P2A, then envelope (Env) (see Figure 2 and Andersson et al. [16]). We did this realizing that the 1:1 stoichiometry provided by the P2A sequence would produce Env in excess of requirements, but previous reports have also suggested that self-surface expressed Env may contribute to immune responses [34]. More troublesome were the reports of past difficulties in making MVA express full length Env, which demonstrated selection for truncations in the cytoplasmic tail of Env during vector propagation [35]. Indeed, the first MVA vaccines encoding HIV and SIV uniformly contained truncations in the cytoplasmic tail of the HIV Env proteins. These truncations were, nevertheless, beneficial for antigen incorporation into VLPs, but with then-unknown consequences for antigenicity [36]. Adenoviruses are known to suffer from the same propagation limitations of certain genes and antigens; however, Cottingham et al. demonstrated that tetracycline repressor-dependent reduction of transgene expression could rescue vectors that were otherwise difficult to produce [37]. Accordingly, a similar system was used to rescue viruses with the intended full-length *env* gene [16] that were clearly capable of producing massive amounts of VLPs in cell types non-permissive for adenovirus replication (see Figure 3 and Andersson et al. [16]). 

## 5. Adenovirus Vectored VLVs Targeting HIV, SIV, and *P. falciparum*

**Figure 4:**
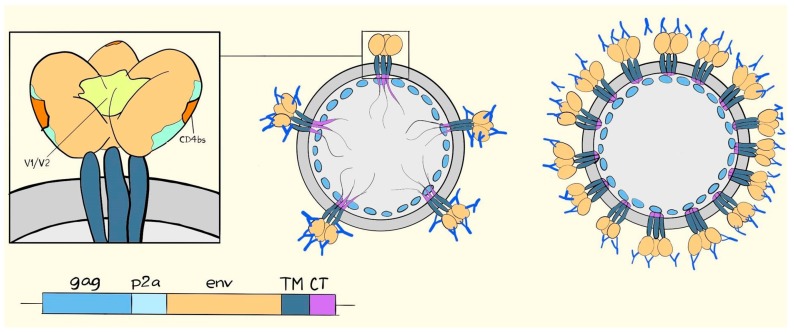
Drawing of the HIV Env trimer and its potential accessibility on VLPs. In the left drawing, Env is depicted with gp120 in light brown and the visible parts of gp41 containing the transmembrane region in dark blue. In the middle drawing, VLPs, scarcely coated with Env, can be seen to be approachable from all angles. Dense coating of the VLPs, as depicted in the right drawing, makes gp120 more accessible relative to gp41.

The inability of the former MVA vectors to propagate a full-length cytoplasmic tail, coupled with published reports on VLP antigen density as a primary determinant of responses against difficult antigens [38], made it logical to study the relation between tail truncations and induced immune responses. Full-length and truncated Env were packaged into SIV Gag-based VLPs, and used as a priming VLVs, before boosting with heterologous adenovirus vectors expressing the full-length Env on VLPs [16]. The immediate consequence of the Env tail truncation was that the Env cell surface display increased dramatically and Env incorporation into VLPs increased moderately. Additionally, the displayed Env conformation became much more accessible to non-neutralizing specificities like those detected with the 17b monoclonal antibody [39]. In fact, even with an increase of up to approximately 40-fold on the cell surface, which could be detected with the 17b or VRC01 monoclonal antibodies, increased binding of the conformation-specific antibodies, PGT145 and PGT151, that require a tightly folded trimer, was not observed [40]. In this light, it is perhaps not surprising that immunization with the truncated version resulted in a significantly stronger antibody response towards the recombinant gp140CFI protein, which is not a tightly folded antigen, and only a small, barely significant increase in binding to trimeric Env purified from VLPs [16]. Conversely, priming with a full-length tail gave responses that trended towards an increased gp41 response and the ratio of gp120/41 binding responses was significantly higher in the truncated tail group [16]. The dramatic changes in Env conformation were consistent with recent results in transfected cells [36] but it makes interpretation of the effects of Env incorporation into VLVs quite difficult. Superficially, one would not think that an excess antigen load, solely consisting of incompletely folded Env, would be beneficial, but this may be a wrong prejudice, as the RV144 trial primed with a poxvirus-delivered VLV essentially must have displayed a gp120 monomer dangling from the VLPs [41]. That the full-length antigen group trended toward higher gp41 responses is interesting, but the mechanism is not known and neither is the significance. One could imagine both reduced steric constraints (illustrated in Figure 4) and reduced competition from the many more accessible gp120 epitopes in the truncated constructs. Certainly, access to epitopes is important as we, in an unpublished cohort, also tested fully transmembrane deleted gp140CFI, which is secreted unbound to the encoded VLPs and which did elicit higher responses towards gp41 (Andersson et al. unpublished). Likely, a part of this response could be mediated by antibodies approaching the gp41 from angles that would be from within the VLPs in the constructs containing the TM region. 

An alternative to binding antibodies would be to look at neutralization, but neither of the VLVs tested in Andersson et al. [16] neutralized tier 2 viruses, and, while neutralization was induced against tier 1 viruses, the different vaccines used for priming did not result in significantly different tier 1 neutralization titers. Unfortunately, transmitted viruses are, for the most part, tier 2 viruses and, thus, the readout can only tell us that we would not be able to neutralize HIV with the kind of responses we obtained in mice. The absence of tier 2 neutralization can be seen as disappointing in comparison to emerging data in the field, but that would be a gross misinterpretation. The response types now known to be inducible by SOSIP BG505 and related to more consistently folded trimeric Env antigens, is limited to autologous responses and highly restricted towards other types [42]. Indeed, these responses typically target rare defects in Env glycosylation or linear non-conserved epitopes [43,44]. While these constructs have clearly solidified our knowledge of the challenges faced in HIV vaccine design, they have provided no clear path forward. Indeed, the focus on obtaining tier 2 virus neutralization may be detrimental, as this draws resources and focus from DNA and virus vectored regimens that, while not able to yield the same consistency in autologous tier 2 neutralization, can provide broader binding antibody responses [45]. Currently, the only inducible mechanism of protection we know of with some certainty is that protection against HIV acquisition can be elicited by broadly cross-reacting antibodies [46]. 

The inability to provide a qualified interpretation regarding the expected efficacy of VLVs generated with full-length and truncated Env displaying VLPs, is typical of the current status of the HIV field. We know that particular flavors of antibodies predicted short-term protection in the RV144 trial, but also that non-human primates respond with different specificities to HIV Env vaccines than humans. This has been demonstrated by simianized vaccines, in which an SIV Env and Gag replaces the HIV sequences in vaccines otherwise identical to the RV144 trial [47]. Nevertheless, these simianized vaccines seem to induce protection against repeated low dose challenges, just as the RV144 trial [48]. Currently, no one knows what the inbred or outbred murine counterpart is of the poorly understood human correlates of protection and it is possible that, rather than specific correlates, functional correlates would translate better between species [49,50]. At present, this is largely speculative and what is really needed is qualified testing in non-human primate models. Such tests have not been a realistic option in the past for HIV, but would now be possible to perform with increased accuracy as a new generation of Simian Human Immunodeficiency Viruses (SHIVs), with transmitted founder-like Env sequences, has become available [51]. 

While the tail truncation succeeded in increasing VLP incorporation as desired, it changed the antigenicity to a large degree. For that reason it could not be answered how the antigen density on particles influenced specificity, quantity, and avidity of responses on an enveloped virus-encoded retroviral VLP. To address this question, we made similar designs of a *P. falciparum* derived antigen VAR2CSA, that is a *Plasmodium falciparum* erythrocyte membrane protein-1 (PfEMP1) family member. VAR2CSA is necessary for the adhesion to placental chondroitin sulfate A, and, thereby, to cause pregnancy-associated malaria [52]. PfEMP1s are assembled from modular domains and, thus, provided the opportunity to use an efficient folding and ligand binding protein not likely to be dependent on signals in the intracellular tail for correct folding [53]. This antigen was directed into the ER using a synthetic signal peptide and anchored with either a mouse mammary tumor virus envelope tail, an influenza A hemagglutinin tail, or was left non-anchored for secretion of antigen [52]. The results were quite clear in that VLP incorporation improved the quantity of the responses after single administration and the functionality of the responses remained superior in the VLP designs after repeated protein boosting. It was also encouraging to notice that the hemagglutinin tail provided better incorporation into VLPs, which was paralleled by more functional responses after adenovirus VLV immunization, as compared with the less efficiently incorporated mouse mammary tumor virus envelope tail. Interestingly, a peptide array used to measure responses against linear B cell epitopes showed more specificities targeted with the hemagglutinin tail anchored antigen [52]. While these data are a poor substitute for an HIV-specific response, they are in agreement with similar studies using DNA-encoded VLPs, which do elicit more potent responses towards VLP-displayed Env, compared to membrane-anchored Env with the same Env sequence [20]. The results, using peptide arrays, also suggested a potential basic mechanism: that efficient VLP incorporation could trigger a more functional and broader response against the displayed antigen [52]. The latter interpretation is also in agreement with other studies showing that VLP incorporation alters the specificity of the induced antibody response [54] and that intrastructural help from T cells specific for VLP proteins, increases the ability of the antibodies to elicit Fc receptor-mediated effector functions [55]. Interesting correlates aside, we currently do not know to what extent protective non-neutralizing antibody responses target the closed conformation of transmitted founders with tier 2 characteristics in vivo or if they, as an alternative example, trigger antibody-dependent cell-mediated cytotoxicity (ADCC) via binding to a subset of misfolded proteins on cell surfaces or virions. Ideally, it should be possible to change the incorporation level of Env without altering antigenicity and, thereby, induce stronger and broader responses against the presumed most relevant Env conformation for tests in primate models. Vaccines with enhanced VLP incorporation, displaying the most relevant closed trimer conformation, could then be compared with vaccines expressing Env with truncated tail conformations for immunogenicity and protection in non-human primate models. Such an experiment could potentially be envisaged using non-native tails, as in our VAR2CSA vaccine, and these tails could be used to display stabilized transmitted founder-like Env proteins, such as BG505 based NFL trimers [56]. 

## 6. Suggested Mechanisms of Antibody-Mediated Protection from SIV, SHIV, or HIV Infection

Only stable trimers, that to a large degree avoid displaying non-neutralizing epitopes associated with open Env conformations, seem capable of inducing tier 2 virus neutralizing antibodies [42]. However, the RV144 trial induced protection, seemingly, via cross-reacting, but non-neutralizing V1V2 antibodies capable of eliciting ADCC. Additionally, the high avidity of Env trimer binding antibodies induced by MVA VLVs, is a protective correlate in SIV and SHIV challenge models [48,57]. The battle between the quest for neutralization and for probing deeper into non-neutralizing antibodies is, however, still unresolved. Certainly, it must be acknowledged that non-neutralizing antibodies, by themselves, are not very efficient [58], but, then again, otherwise protective recombinant Env protein immunogens that are very poor T cell inducers have been able to induce cross-protective antibodies in animal models, even though such immunity was not transferable by serum from protected animals [59]. The question of antibody localization comes to mind and mucosal antibodies have, indeed, been correlated with protection from SIV [60]. Many studies have also found correlates of protection with antibodies neutralizing tier 1 viruses and so-called “sieve” analysis, which compares neutralization sensitivity of pre-challenge viral swarms with transmitted viruses, also points to neutralization as a key determinant of partial efficacy [61]. These latter types of studies are, however, prone to erroneous conclusions for this particular question, as non-neutralizing antibodies, per definition, bind open conformations that are shielded in neutralization resistant viruses [62]. 

## 7. VLV Induced Antibodies in Prime-Boost Regimens

One of the most impressive findings in this era of heterologous prime-boost regimens, has been the observation of consistent induction of Env trimer binding antibodies in animals vaccinated three times with homologous MVA vectored VLVs. Such protection is, as highlighted above, correlated with high avidity antibodies towards Env and not T cell responses, and although the specificity is changed to gp41, similarly highly avid antibody responses can be induced in healthy human volunteers [30]. To substantiate the point that protection in this system is antibody mediated, the T cell response towards the MVA vaccines is rather limited, and reducing the T cell response further in vaccinia virus immune animals [63] or increasing it by DNA prime [64], has no effect on post-exposure control of viremia that cannot be explained by differences in the antibody response.

The robust induction of antibody responses towards MVA VLVs stand in contrast to a recent trial using BG505 Env sequences in MVA vectors in rabbits, where the induction of Env trimer binding antibodies was unreliable, and generally much inferior to repeated recombinant protein or adenovirus vector immunization, which yielded robust responses even after the first immunization [45]. Importantly, combinations of adenovirus prime and MVA boost yielded potent responses after the first booster immunization, in agreement with numerous other studies. This recent study could be interpreted to indicate that VLVs based on an HIV Env and Gag are superior in a single formulation; however, it must be emphasized that the differences can also be attributed to the different Env antigen (BG505) [45] vs. SIV mac239 [23,64] vs. HIV clade B [16,65], and not just the existence of a VLP in the SIV and HIV clade B VLV studies [65]. 

In summary, VLVs seem, at least, competitive and, perhaps, superior for the induction of antibody responses compared to secreted formulations [52], but it has been hard to substantiate superiority in the HIV field, where the antigenicity of Env is highly affected by the strain of the antigen and the exact formulation of the VLV. Indeed, we do not know what a good and realistic antibody response is. Controlled trials against realistic SHIV models with transmitted founder-like Env sequences are urgently needed.

## 8. VLVs as Inducers of T Cell Responses

While the MVA VLVs mentioned above [57,65]—in contrast to the non-VLV MVA expressing BG505 Env [45]—are efficient inducers of trimer binding antibodies, they are poor T cell inducers [64]. In agreement with other studies, MVA VLVs need DNA priming [64] or, ideally, another viral priming [45], to facilitate competitiveness of the transgene product with vector derived products [66]. 

Replication-defective adenovirus vectors, in contrast, are capable inducers of T cell responses against a number of transgenes, as the commonly used E1 deletion reduces the expression of vector proteins outside the viral producer cells [25,67]. In combination with MVA used as a booster vaccination, the adenovirus primed T cell responses have given rise to some of the most impressive T cell frequencies in both human and animal models [3,33]. Adenovirus-primed and, in particular, adenovirus-boosted T cell responses against the structural Gag antigen do not seem capable of preventing any fraction of HIV/SIV infections, but they are clearly the most validated cells for post-acquisition control of SIV infection [32,68]. Even in the human STEP trial, which failed in its overall objectives, those vaccinees who succeeded in raising multiple T cell responses towards Gag had significant and long-term reductions of viremia [69]. 

In NHP studies, a number of T cell correlates have been proposed and it seems efficacy in controlling infection is more a matter of inducing the right specificities than inducing many specificities. Obviously, a broad response will be more likely to include protective epitopes, but it is important to realize that many T cell responses are without any inhibitory impact on viral replication. Vaccination exclusively against protection-associated epitopes, compared to vaccination exclusively against non-protection-associated epitopes, showed that the epitopes not associated with long-term protection were completely without effect, even though they could induce responses of equal magnitude [70]. This kind of effect can potentially be related to the ability of mutations, in such epitopes, to confer a replicative penalty on the virus, as suggested in human correlative studies [71].

**Figure 5:**
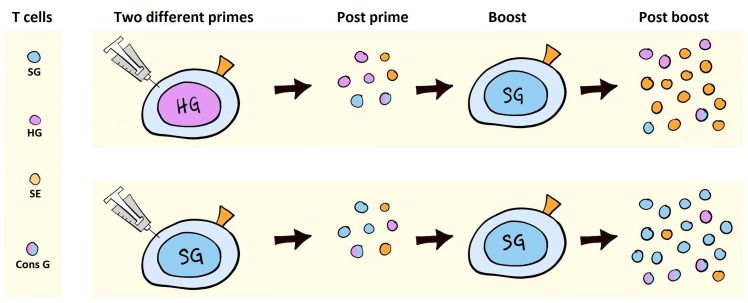
Graphic illustration of the results obtained in Andersson et al. 2016 [46]. The left panel depicts the T cells with specificity for SIV Gag (SG), HIV Gag (HG), SIV Env (SE), or with dual specificity for HIV and SIV Gag (Cons G). The priming expands the T cells with relevant specificities (mid-left). Booster immunization results in the expansion of SIV Env-specific T cells when heterologous gag is applied, and the expansion of SIV gag, including cross-reactive Gag-specific T cells, when a homologous Gag immunization is applied. Thus, in the face of Env antigen competition, boosting of cross-reactive Gag-specific T cells is inhibited.

SIV VLVs have been used in an attempt to induce an immune response focused on the conserved elements (CEs) of Gag, by varying the origin of the Gag sequence (HIV and SIV mac239) between prime and boost immunization [23]. This was done with the *a priori* hypothesis that a heterologous Gag sequence would boost conserved epitopes selectively, but, rather surprisingly, changing the Gag sequences between an adenoviral VLV prime and a MVA VLV boost diminished boosting of Gag-specific responses and resulted in immunodominance of the SIV Env sequence that was shared in the prime and boost vector. In contrast, animals boosted with the same SIV Gag in the adenovirus and MVA VLVs exhibited very strong SIV Gag-specific responses, which were superior to the heterologous Gag group, both within the cross-reactive CE-specific responses and overall (see schematic outlining the apparent outcome in Figure 5). Thus, from the perspective of a normal vaccine design, the heterologous VLV design did everything it was supposed to do, except enabling the selective stimulation of cross-reactive epitopes between the prime and boost immunization. These results differ from a number of other studies in the field, in both mice and humans [72,73,74]. Thus, Barouch and co-workers did not find any appreciable differences between consensus and a circulating HIV clade in prime-boost regimens, but in these studies no Env was encoded, and the differences between the Gag sequences were in the same 90% similarity range, where we generally observe reliable cross-reactivity [72,75]. Conversely, the human studies using either Env or Gag of more than 10% sequence diversity have, indeed, elicited improved cross-reactive responses [73,74], suggesting that our experiment could have worked, if not for the immunodominance exhibited by the homologous Env sequence in our study [23]. At first glance, the critical role of immunodominance hierarchies during prime-boost VLV immunization regimens may appear to be a limitation of the VLV design, but it is also an opportunity. HIV Envs are considerably more diverse than the corresponding Gag sequences, and it should be possible to design prime-boost regimens with more diversity in the Env than in the Gag component of VLVs. Furthermore, for eliciting immune responses matching protective correlates against HIV, we are interested in combining cross-reactive antibody responses against Env, cross-reactive T cell responses against Gag, and, potentially, other conserved epitopes residing in the structural genes [76]. Importantly, T cells do not, in general, appear to exert immunodominance over Env-specific antibody responses, rather both CD4^+^ and CD8^+^ T cells enhance antibody responses through a phenomenon called intrastructural help [55,77]. In Andersson et al. [23], a similar positive correlation was observed between the CD8^+^ T cell response towards Gag in the homologous Gag group and the CD8^+^ T cell response towards Env in the heterologous Gag group [23]. The phenomenon of concomitant T cell immunodominance and assistance towards antibody responses could also have been responsible for the previously reported increased response towards MVA VLVs in MVA-immune animals. Here, transgene-specific cellular responses were seen to be blunted (presumably by vector-specific immunity), but antibody responses and, in particular, their functionality were increased [63]. 

Accordingly, it should be possible to select for broad antibody responses and avoid or exploit immunodominance of non-crossreactive Env or Gag-specific T cells by varying the sequences between prime and boost, but the proof of this potential vaccination protocol awaits ongoing studies. One of the key uncertainties that have become apparent from attempts to elicit cross-reactive T cells from heterologous insert prime-boost VLVs, is whether avoiding competition is sufficient to select for the desired specificities. The sequences associated with protection from HIV are also selected through evolution to be poorly immunogenic in the most common human haplotypes [78,79]. Evidence suggests that conserved elements can be primed by DNA and then boosted with full-length Gag sequences encoded in adenoviral vectors, but such an approach adds yet another layer of complexity to a future HIV vaccine [80]. If it is possible to express similar concatenated epitopes, either by co-encoding them or inserting them in the VLPs encoded by viral vectors, and raise immune responses to such epitopes, is presently unknown. Viral vectors hold several advantages over the tested DNA vaccines, particular with regard to immune potency, but they do come with a number of viral epitopes that can exert immunodominance over subdominant epitopes [81,82]. Alternatives also exist by looking at other viral genes, such as *nef* and *pol*, where there are conserved epitopes, of which some are protection-associated; however, Nef is quite variable over most of the sequence and Pol is likely to be less immunogenic at low viral replication levels [83,84]. Therefore, a response to such conserved epitopes might be easier to induce than a response to the conserved epitopes embedded in Gag; although, to insert them into vaccines would also run the risk that they are less effective in controlling virus replication to low levels. 

If these issues could be adequately resolved in future works, VLVs would be capable of inducing broad binding antibody responses, potent T cell responses, and selection for recognition of structurally conserved epitopes. Such properties summarize the current knowledge on natural and vaccine inducible immunity towards HIV and show that the test of such a vaccine would be a highly relevant experiment.

## 9. Conclusions

Virus-like-vaccines are virus vectored vaccines that infect APCs and tissue resident cells to initiate strong cellular immune responses, and then lead to the secretion of virus-like-particles within the vaccine recipient. This enables the production of structurally relevant antigens to stimulate B cells and provides extracellular antigens in an inflammatory milieu to stimulate helper T cells and further CD8^+^ T cell responses through cross-presentation. VLVs are potent vaccination tools because they deliver the antigen in the form to which the immune system is developed to react. Current experience has provided the best results against HIV using a VLV [2] and a new category of VLVs are capable of reproducing the currently available correlates of vaccine protection against HIV in animals [23,48].
